# Adaptive laboratory evolution of a thermophile toward a reduced growth temperature optimum

**DOI:** 10.3389/fmicb.2023.1265216

**Published:** 2023-10-12

**Authors:** Maria Lehmann, Christoph Prohaska, Benjamin Zeldes, Anja Poehlein, Rolf Daniel, Mirko Basen

**Affiliations:** ^1^Department of Microbiology, Institute of Biological Sciences, University of Rostock, Rostock, Germany; ^2^Genomic and Applied Microbiology and Göttingen Genomics Laboratory, Georg-August University, Göttingen, Germany

**Keywords:** adaptive laboratory evolution, origin of life, cold adaptation, acetogens, thermophiles, *Thermoanaerobacter kivui*

## Abstract

Thermophily is an ancient trait among microorganisms. The molecular principles to sustain high temperatures, however, are often described as *adaptations*, somewhat implying that they evolved from a non-thermophilic background and that thermophiles, i.e., organisms with growth temperature optima (T_OPT_) above 45°C, evolved from mesophilic organisms (T_OPT_ 25–45°C). On the contrary, it has also been argued that LUCA, the last universal common ancestor of *Bacteria* and *Archaea*, may have been a thermophile, and mesophily is the derived trait. In this study, we took an experimental approach toward the evolution of a mesophile from a thermophile. We selected the acetogenic bacterium *T. kivui* (T_OPT_ 66°C) since acetogenesis is considered ancient physiology and cultivated it at suboptimal low temperatures. We found that the lowest possible growth temperature (T_MIN_) under the chosen conditions was 39°C. The bacterium was subsequently subjected to adaptive laboratory evolution (ALE) by serial transfer at 45°C. Interestingly, after 67 transfers (approximately 180 generations), the adapted strain Adpt45_67 did not grow better at 45°C, but a shift in the T_OPT_ to 60°C was observed. Growth at 45°C was accompanied by a change in the morphology as shorter, thicker cells were observed that partially occurred in chains. While the proportion of short-chain fatty acids increased at 50°C vs. 66°C in both strains, Adpt45_67 also showed a significantly increased proportion of plasmalogens. The genome analysis revealed 67 SNPs compared to the type strain, among these mutations in transcriptional regulators and in the cAMP binding protein. Ultimately, the molecular basis of the adaptation of *T. kivui* to a lower T_OPT_ remains to be elucidated. The observed change in phenotype is the first experimental step toward the evolution of thermophiles growing at colder temperatures and toward a better understanding of the cold adaptation of thermophiles on early Earth.

## 1. Introduction

Temperature is a key environmental factor that determines the biodiversity and species composition of habitats as it requires specific traits to allow organisms to thrive at distinct temperatures. The temperature range of habitats on Earth is huge, and living cells have been found from the polar environments and high-altitude regions (De Maayer et al., 2014) to terrestrial mud springs and submarine hot vents (Stetter, 2006). The growth temperature profile of every species is characterized by the cardinal temperatures, the minimal (T_MIN_), the maximal (T_MAX_), and the optimal temperature (T_OPT_) that allow growth (Wiegel, 1990). Organisms adapted to extremely cold or hot temperatures, or temperature extremophiles, are classified by their T_OPT_. In contrast to mesophiles growing at more or less ambient temperatures (T_OPT_ 20–45°C), psychrophiles prefer a T_OPT_ lower than 15°C (De Maayer et al., 2014). Thermophiles thrive optimally at temperatures higher than 45°C, while organisms with a T_OPT_ >65–70°C are called extreme thermophiles, and hyperthermophiles possess a T_OPT_ of >80°C (Wiegel, 1990; Zeldes et al., 2015). The temperature range of individual thermophiles may be very broad (Wiegel, 1990). For example, the hyperthermophilic Archaeon *Pyrococcus furiosus* grows optimally at 100°C, with a T_MAX_ of 103°C and a T_MIN_ of 65°C (38 K; Fiala and Stetter, 1986), and the methanogen *Methanothermobacter thermautotrophicus* even has a span of 55 K (Wiegel, 1990).

The specific adaptations of thermophiles have been extensively studied and include the modification and protection of all types of cellular macromolecules. For example, DNA in (hyper)thermophiles is protected by (positively charged) polyamines and by histones in Archaea (Imanaka, 2011). Common features seen in proteins of thermophiles are increased van der Waals interactions, hydrogen bonds, ionic interactions to stabilize the secondary or tertiary structure of the protein, a more hydrophobic interior, and an increased packing density of the protein (Berezovsky and Shakhnovich, 2005; Imanaka, 2011). The folding of proteins in thermophiles is supported by the action of particular chaperones, and proteins and DNA are stabilized within the cell by compatible solutes (Imanaka, 2011). Mechanisms to stabilize the cytoplasmic membrane include ether-linked membrane lipids in archaea, while the degree of saturation and the amount of branched-chain and longer fatty acids increase with temperature in bacteria (Siliakus et al., 2017). Despite these and many other specific traits attributed to life at high temperatures, a comprehensive understanding of “thermophily” is still lacking (Canganella and Wiegel, 2014), particularly toward its evolution. On the one hand, the aforementioned molecular traits to thrive at high temperatures may be derived, having evolved from a mesophile background. On the other hand, these traits may be ancient, and mesophiles may have evolved from ancient thermophiles. For example, life may have emerged at moderately warm hydrothermal and alkaline vent fields such as Lost City (40–90°C; Kelley et al., 2005). This environment is cold enough to allow first (bio)molecular reactions but rich in trace elements and in H_2_ and CO_2_. These gaseous substrates may have supported the formation of biomass precursors coupled with energy conservation, as found in recent acetogenic bacteria and methanogenic archaea (Martin, 2020). Interestingly, to the best of our knowledge, the hypothesis that mesophiles are derived from thermophiles has never been tested in laboratory evolution experiments.

**Figure 1:**
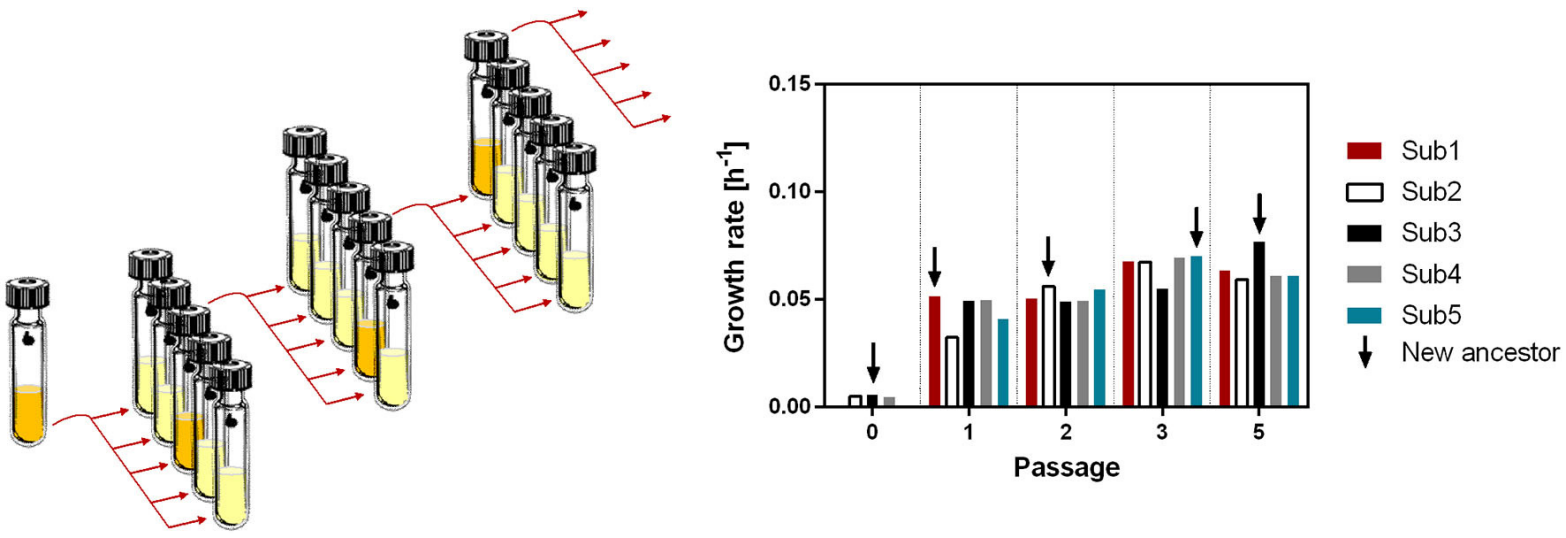
Serial passaging modified after Strauss et al. (2019). Five subpopulations of *T. kivui* were incubated for 24 h in a complex medium containing 25 mM glucose as a carbon source. The subpopulation with the highest increase in OD_600_ was used to generate five new subpopulations in a fresh medium.

In this study, we aimed to evolve the bacterium *Thermoanaerobacter kivui* as acetogen with a T_OPT_ of 66°C is capable of growing in environments comparable to Lost City, and where the first life may have emerged, toward growth at lower temperatures. *T. kivui* has been described as an acetogen growing on a variety of substrates, including H_2_+CO_2_ but also on the sugars glucose, mannose, and fructose (Leigh et al., 1981). The physiology of *T. kivui* has recently been studied to a larger extent. For example, it has been shown to grow on the sugar alcohol mannitol (Moon et al., 2019), in bioelectrical systems on anodes (Deutzmann et al., 2022), and it has been subjected to ALE to grow on carbon monoxide (Weghoff and Müller, 2016). Genetic tools for genome modification (Basen et al., 2018) and plasmid-based protein production (Katsyv et al., 2021) were developed, allowing to study one of its energy-conserving hydrogenases (Katsyv and Müller, 2022), and the soluble hydrogen-dependent carbon dioxide reductase (Jain et al., 2020; Dietrich et al., 2022). This prompted us to pick *T. kivui* as a model thermophilic acetogen for ALE experiments. We grew five populations of *T. kivui* DSM2030 at 45°C (21 K below T_OPT_) and used the fastest growing population as inoculum for the next five populations cultivated at 45°C (Figure 1). After 67 serial transfers, corresponding to ~180 generations, we studied the phenotype and the genotype of the evolved strain, Adpt45_67.

## 2. Materials and methods

### 2.1. Bacterial strains and cultivation

*Thermoanaerobacter kivui* strain LKT-1 (DSM2030), referred to as the type strain, and the adapted strain Adpt45_67 were cultivated under strict anoxic conditions at different temperatures between 40°C and 66°C in complex or defined media as described previously (Weghoff and Müller, 2016; Basen et al., 2018). Complex media contained Na_2_HPO_4_ × 2 H_2_O, 50 mM; NaH_2_PO_4_ × 2 H_2_O, 50 mM; K_2_HPO_4_, 1.2 mM; KH_2_PO_4_, 1.2 mM; NH_4_Cl, 4.7 mM; (NH_4_)_2_SO_4_, 1.7 mM; NaCl, 7.5 mM; MgSO_4_ × 7 H_2_O, 0.37 mM; CaCl_2_ × 2 H_2_O, 42 μM; Fe(II)SO_4_ × 7 H_2_O, 7.2 μM; KHCO_3_, 54 mM; cysteine-HCl × H_2_O, 3 mM; resazurin, 4.4 μM; 0.2% (w/v) yeast extract, 10 ml/l of trace element solution DSM141, and 10 ml/l of vitamin solution DSM141. Defined media were prepared similarly as complex media without the addition of yeast extract. The medium was flushed with N_2_:CO_2_ (80:20 [v:v], 1.1 x 10^5^ Pa) before autoclaving. The pH of the medium was 7.5 after flushing. The agar medium was supplemented with 1.5% Bacto agar (BD Difco, BD Life Sciences, Heidelberg, Germany). All gases were purchased from Westfalen AG (Münster, Germany).

Growth experiments were carried out in 20 ml Hungate glass tubes with 5 ml of medium or in 100-ml or 200-ml serum bottles with 50 ml or 100 ml of medium, respectively. The glass tubes or serum bottles were sealed with butyl rubber stoppers under an atmosphere of N_2_:CO_2_ (80:20 [v:v], 1.1 x 10^5^ Pa) unless denoted otherwise (Basen et al., 2018). Glucose was added as a carbon source from a sterile anoxic stock solution to a final concentration of 25 mM. If H_2_ + CO_2_ were used as substrates, tubes were only filled with medium to one-fourth of the volume, and the remaining headspace was replaced with H_2_:CO_2_ (80:20 [v:v], 2 x 10^5^ Pa). For growth experiments of *T. kivui* at different temperatures, initial cultures were grown at 66°C to an OD_600_ of 1 and diluted 1/10 in fresh medium, followed by incubation at the desired temperature. Plating and cultivation of solid media were carried out according to Basen et al. (2018). The agar dilution series and the isolation of single colonies from it were performed according to Widdel and Bak (1992).

### 2.2. Monitoring growth, cell morphology, and metabolites

Growth in the liquid medium was monitored by measuring the optical density at 600 nm (OD_600_) with a spectrophotometer. Cell morphology was documented using a Nikon Eclipse Ni-U microscope equipped with a Nikon DSFi3 camera (Nikon, Tokyo, Japan). Glucose and organic acid concentrations were determined by HPLC as described previously (Zeldes et al., 2023).

### 2.3. Adaptative laboratory evolution

The ALE approach by serial passaging was performed as described by Wein and Dagan in Strauss et al. (2019). In brief, *T. kivui* DSM2030 was grown on agar plates at 66°C. Three colonies were randomly sampled as the ancestral clones. The serial passage experiment was initiated by inoculating the three clones in Hungate tubes containing 2 ml of complex medium and incubating at 45°C. Subsequently, the ancestral populations were sampled into five subpopulations of each replicate. The five subpopulations were grown at 45°C, and growth was monitored as described. The subpopulations of each replicate having the highest increase in OD_600_ after 24–28 h were selected as the ancestors for the next subpopulations.

### 2.4. Single-nucleotide polymorphism analysis

The genomes of adapted strains were analyzed for single-nucleotide polymorphisms as recently described (Zeldes et al., 2023).

### 2.5. Analysis of cellular fatty acids

*T. kivui* cultures were grown as described to an OD_600_ of approximately 1. Cultures were harvested for 5 min at 5,000 × *g* and 4°C. Cell pellets were suspended in 0.7% (w/v) aqueous MgSO_4_ and centrifuged again. Cell pellets were freeze-dried, and ~40 mg of cell biomass (dry weight) was sent for fatty acid (FA) analysis at the Deutsche Sammlung von Mikroorganismen und Zellkulturen (DSMZ) in Braunschweig, Germany.

### 2.6. Statistical analysis

Datasets were analyzed by two-way ANOVA with Tukey's test using the software Graph Pad Prism Version 6.01 (GraphPad Software, Boston, USA).

## 3. Results

### 3.1. Growth of *T. kivui* at suboptimal temperatures

**Figure 2:**
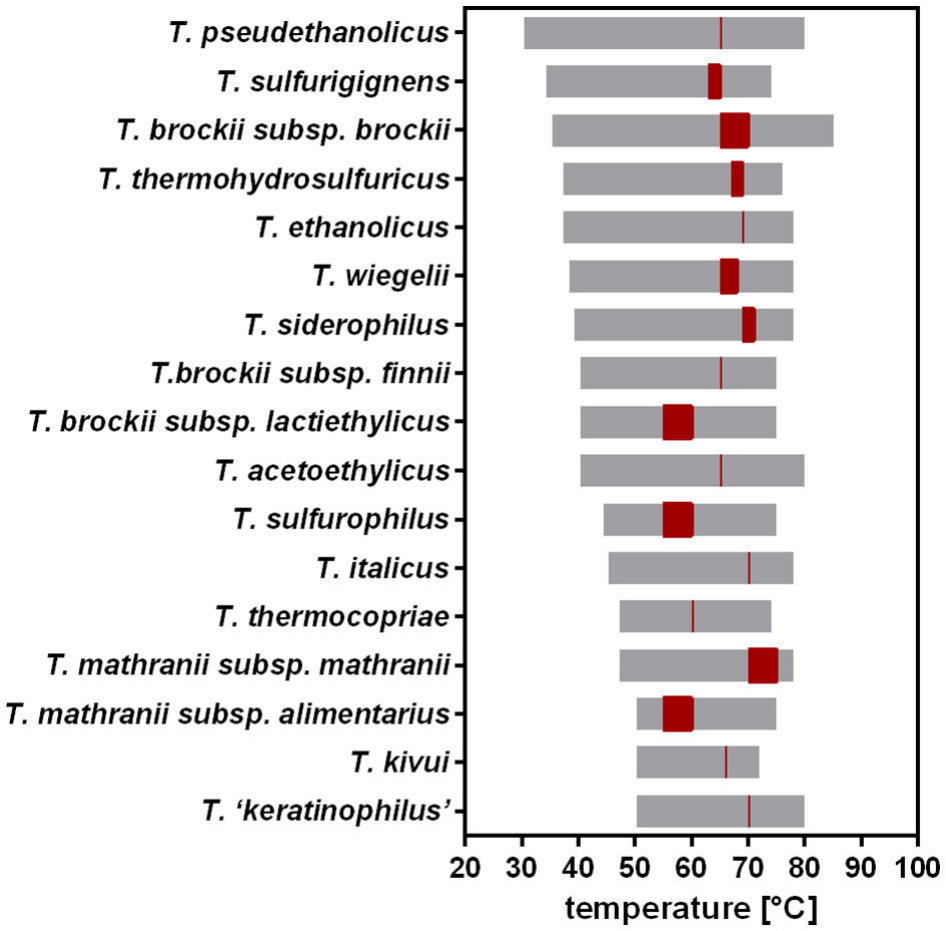
Previously reported temperature ranges and optimal growth temperatures for different *Thermoanaerobacter* species. Temperature range, gray; T_OPT_, red. Data from Onyenwoke and Wiegel (2015).

**Figure 3:**
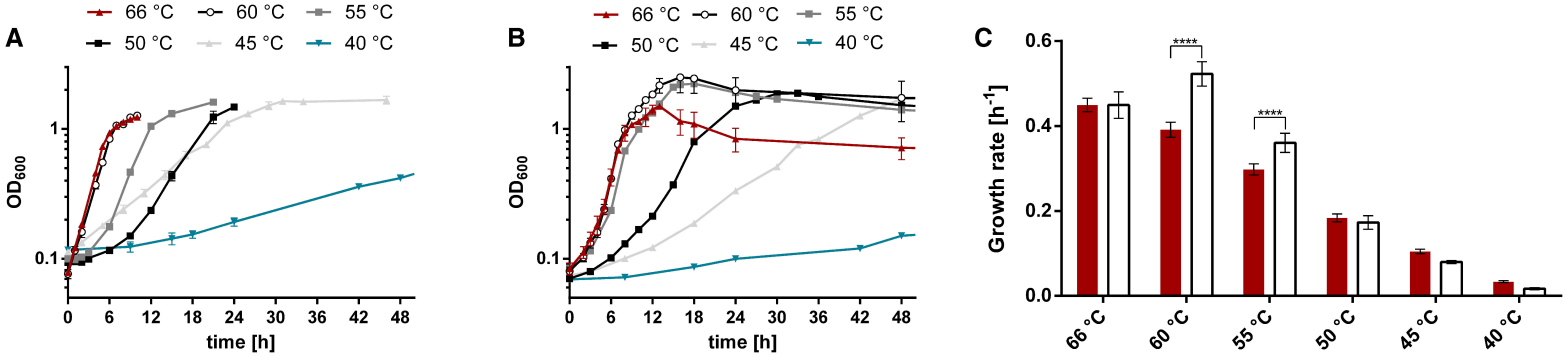
Growth of *T. kivui* strains **(A)** DSM2030 (type strain) and **(B)** Adpt45_67 at different temperatures, and **(C)** the corresponding growth rates. Cells were grown in 50 ml complex medium in 100 ml serum bottles with 25 mM glucose as a carbon source. Red triangles, 66°C; black open circles, 66°C; gray squares, 55°C; black squares, 50°C; gray triangles, 45°C; turquoise inverted triangles, 40°C. The error indicated shows the standard deviation from four biological replicates. Significant statistical differences (***p < 0.0005, two-way ANOVA with Tukey's test) between the type strain and the adapted strains at each temperature condition are shown.

*T. kivui* was originally described to thrive at 50–72°C (Leigh et al., 1981). Compared to other *Thermoanaerobacter* species, this is a relatively narrow range (Onyenwoke and Wiegel, 2015), since, e.g., *T. pseudethanolicus* grows between 30°C and 80°C (Figure 2). The T_OPT_ of *T. kivui* (66°C) compares favorably with that of other *Thermoanaerobacter* species (55°C−75°C); however, its reported T_MIN_ of 50°C is among the highest, with many species able to grow at significantly lower temperatures between 30°C and 40°C. Wiegel also reported that they were able to cultivate *T. kivui* at 35°C (1990); however, growth parameters and culture conditions were not specified in the articles. These hints that the published T_MIN_ may be too high prompted us to test the growth of *T. kivui* at suboptimal temperatures. Toward that, we selected a complex medium with a reduced yeast extract content (Basen et al., 2018), and glucose as a substrate over H_2_+CO_2_, since the growth rates and final optical densities in a defined medium and under autotrophic conditions are lower (Jain et al., 2020; Moon et al., 2020). Growth experiments within a temperature range of 40–66°C were performed (Figure 3A). Consistent with the literature, 66°C is the T_OPT_ for the type strain *T. kivui* DSM2030 (Leigh et al., 1981), at a specific growth rate of 0.45 h^−1^ (Figure 3 and Supplementary Table S1). Lower temperatures resulted in decreased growth rates, as expected. The growth of *T. kivui* was monitored at temperatures below 50°C, and slow growth (0.033 h^−1^) was observed at 40°C. At temperatures below 39°C, *T. kivui* did not grow in our hands, in contrast to the report of Wiegel (1990); however, as described above, the author may have used different cultivation conditions. Corresponding to lower growth rates, the maximal OD_600_ as an indicator of the biomass yield was reached after longer incubation periods. For example, an OD_600_ >1 was reached after 7 h at 66°C and 60°C, after 12 h at 55°C, and after 23 h at 45°C. At a growth temperature of 40°C, an OD_600_ of 1 was not reached even after incubation for 108 h (Figure 3A).

### 3.2. Adaptive laboratory evolution of T. kivui to lower temperatures

We decided to use 45°C as the temperature for serial passaging of cultures for ALE, since it seems to provide a good balance of strong selective pressure (only a few degrees above the lowest temperature at which growth is observed) and fast enough growth to achieve a high number of generations through daily passaging. In ALE, organisms are observed under controlled laboratory conditions and specific growth conditions for extended periods of time, allowing the isolation of adapted phenotypes. Many different strategies for ALE have been developed and further improved (Dragosits and Mattanovich, 2013; Strauss et al., 2019), from simple long-term passaging experiments with *Escherichia coli* over several thousand generations (Cooper and Lenski, 2000), to ALE using increased mutation rate and targeted genetic engineering (Luan et al., 2013; Badran and Liu, 2015; Suzuki et al., 2015; Kang et al., 2019). In this study, a variant of the serial passaging strategy (Strauss et al., 2019) was selected, in which five populations were evolved in parallel to increase the number of genotypes tested (Figure 1). Targeted selection was performed continuously on the fastest growing population, defined by the highest increase in OD_600_ within a growth period of 24–28h. Within that time frame, *T. kivui* was still in the exponential growth phase, excluding a more complex adaptation to the stationary growth phase (Vasi and Lenski, 1999). After ~180 generations (passage 67—Supplementary Figure S3), the *T. kivui* population with the highest biomass increase (adapted strain Adpt45_67) was again selected, and a detailed study of the phenotype was performed.

### 3.3. The evolved strain *T. kivui* Adpt45_67 has a lower optimal growth temperature

We compared the growth of the type strain (DSM2030; acclimated, three transfers at 45°C) and the adapted strain Adpt45_67 (~180 generations at 45°C) during growth on the “adaptation” medium, complex medium plus 25 mM glucose, at temperatures between 40°C and 66°C (Figure 3B). We found that under these conditions, Adpt45_67 grows as fast as the type strain at 66°C (0.45 h^−1^ vs. 0.45 h^−1^, Figure 3C, Supplementary Table S1). At 45°C, the temperature chosen for adaptation, Adpt45_67, unexpectedly does not grow faster than the type strain (0.08 h^−1^ vs. 0.104 h^−1^). At 40°C, the lowest temperature tested, growth rates were reduced to below 10% of the rates at 66°C (0.017 h^−1^ and 0.033 h^−1^), demonstrating the challenge of ALE at lower temperatures. Interestingly, the only significant changes were observed at 60°C and 55°C. At both temperatures, Adpt45_67 grew significantly faster than the type strain (0.523 h^−1^ vs. 0.391 h^−1^ at 60°C and 0.360 h^−1^ vs. 0.297 h^−1^ at 55°C), leading to a shift in the T_OPT_ from 66°C to 60°C (see also Supplementary Figure S1).

**Figure 4:**
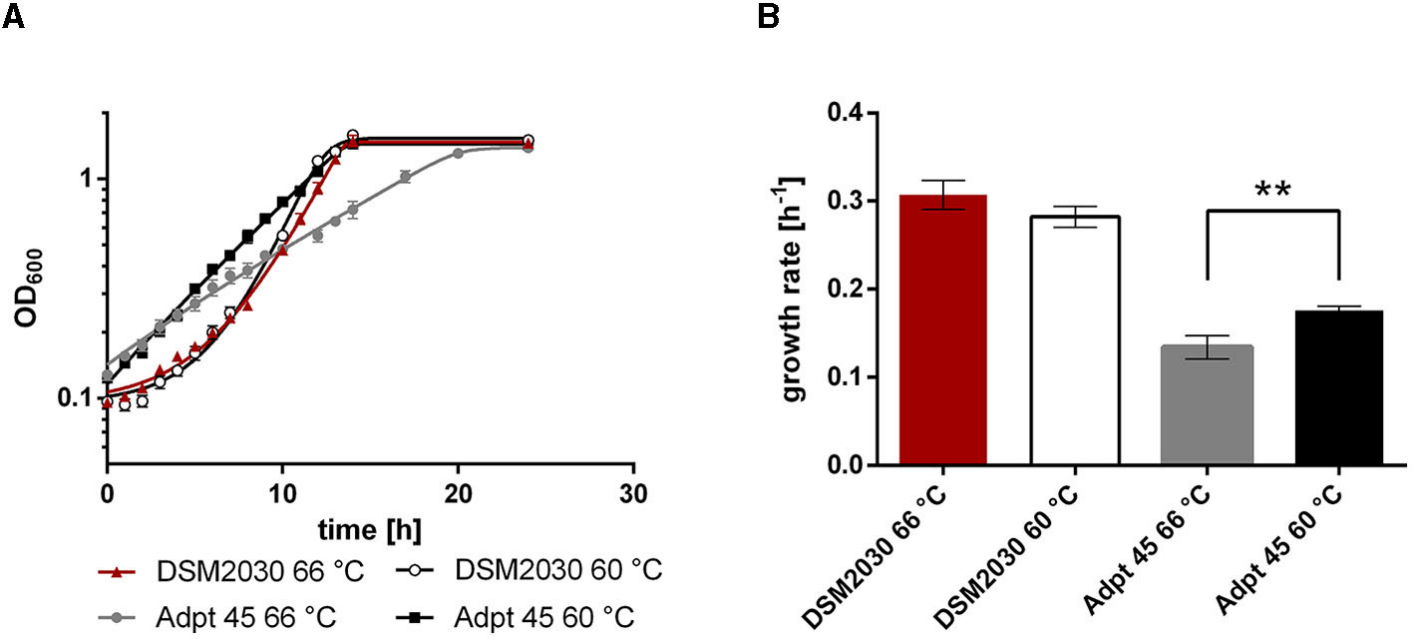
Growth of *T. kivui* strains **(A)** DSM2030 (type strain) and Adpt45_67 in the defined medium at 66°C and 60°C, and **(B)** the corresponding growth rates. Red, type strain at 66°C; white, Type strain at 60°C; gray, Adpt45_67 at 66°C; black, Adpt45_67 at 60°C. Cells were grown in 50 ml of medium in 100 ml serum bottles with 25 mM glucose as a carbon source. The error indicated shows the standard deviation from four biological replicates. Significant statistical differences (**p < 0.005, two-way ANOVA with Tukey's test) between the type strain and the adapted strains at each temperature condition are shown.

We then tested whether the effect is medium-dependent, since adaptations to medium types and compounds have been reported from the Lenski lab (Cooper and Lenski, 2000). We grew the type strain and Adpt45_67 at 66°C and 60°C in a defined medium, which is identical to the complex medium except for the omission of yeast extract (2 g l^−1^). Since *T. kivui* is an acetogen, we initially confirmed that Adpt45_67 still grows on H_2_+CO_2_, forming acetate. Since all ALE experiments and growth rate comparisons (Figures 1, 3) were performed under heterotrophic growth conditions, we again used glucose as a substrate for the comparison of the growth phenotypes. We observed that Adpt45_67 has a significantly lower growth rate than the type strain (Figure 4) at either temperature without yeast extract. This phenotype in the non-adaptive environment was somewhat surprising, since in *E. coli* this has not been observed in short-term evolution experiments (~800 generations) (Kang et al., 2019), but fitness tradeoffs were rather observed in long-term ALE (Leiby and Marx, 2014). However, the observed decreased fitness in the non-adaptive condition (defined medium) described here may be the result of a single or few mutations rather than accumulated mutations, or of a single mutation that favors growth at low temperatures but not in the defined medium. Adpt45_67 still grew without yeast extract and maintained the ability to synthesize amino acids and putative other growth factors. Second, Adpt45_67 grew better at 60°C (0.176 h^−1^) than at 66°C (0.134 h^−1^), confirming the observed shift in T_OPT_ from 66°C to 60°C.

### 3.4. Changes in morphology, fatty acid composition, and in the genome of T. kivui Adpt45_67

**Figure 5:**
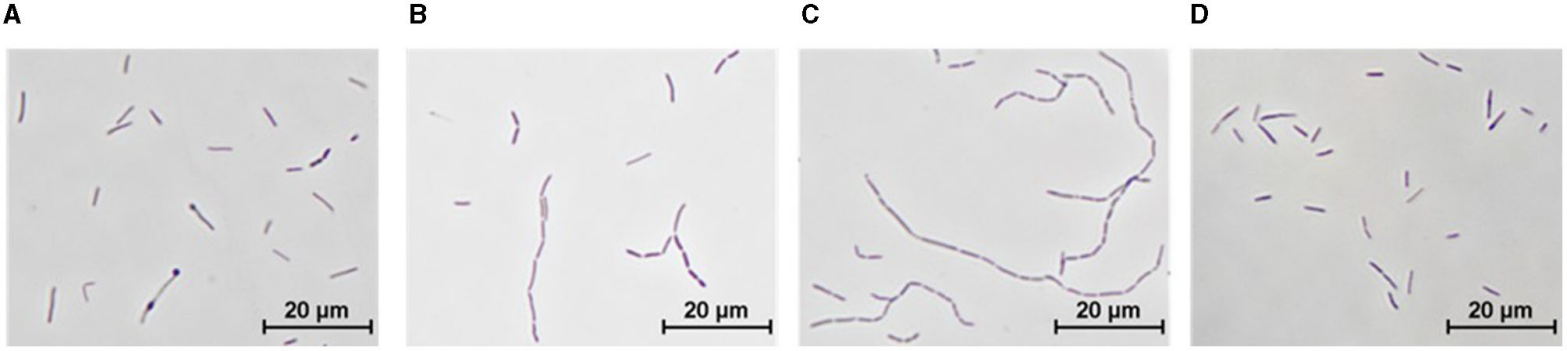
Morphology of *T. kivui* strains as observed by phase-contrast microscopy. *T. kivui* strains were grown in 50 ml of complex medium in 100 ml serum bottles with 25 mM glucose as a carbon source. **(A)** DSM2030 (type strain), 20 h at 66°C (T_OPT_), **(B)** DSM2030 after five transfers at 45°C, 24 h at 45°C, **(C)** Adpt45_67, 24 h at 45°C, and **(D)** Adpt45_67, 24 h at 60°C (T_OPT_).

The type strain of *T. kivui* DSM2030 is characterized by long slender rod-shaped cells during growth on glucose of a size of ~0.7 μm in width and up to 7.5 μm in length (Leigh et al., 1981), which we similarly observed after growth at 66°C for 20 h in the type strain (Figure 5A). Growth of the type strain and the Adpt45_67 at 45°C resulted in an increase in cell diameter and a shortening of cell length to 2–3 μm, as well as a striking number of long cell chains (Figures 5B, C). Interestingly, this morphology appeared to be reversible, since the incubation of Adpt45_67 at 66°C for 20 h resulted in a type strain such as morphology (Figure 5D).

**Figure 6:**
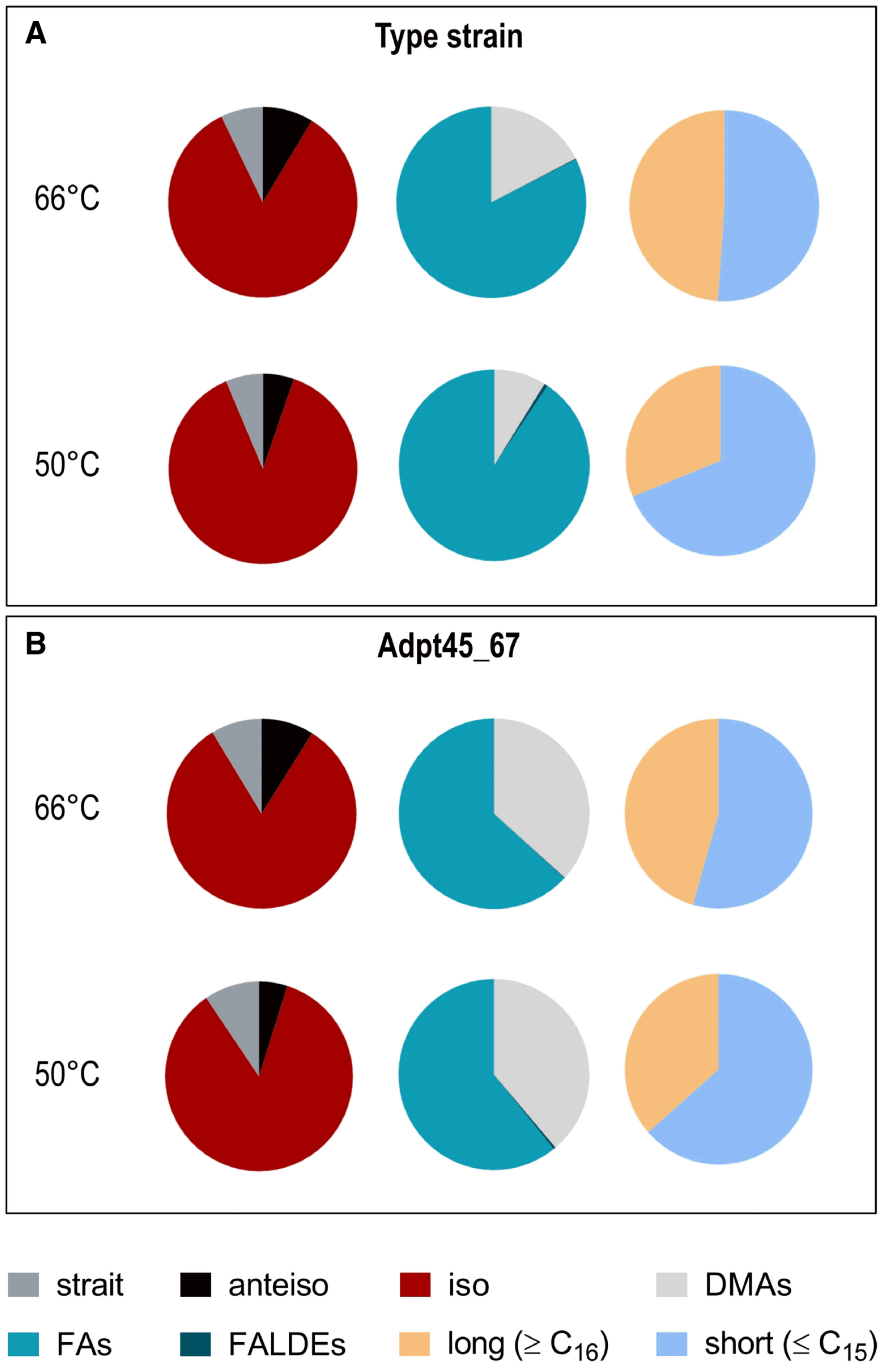
Effect of growth temperature on the total fatty acid composition of *T. kivui*. *T. kivui* strains DSM2030 **(A)** and Adpt45_67 **(B)** were grown in a complex medium with 25 mM glucose at optimal (66°C and 60°C) and suboptimal (50°C) temperatures to an OD_600_ of ~1. Left panels: red, iso-branched; gray, anteiso-unbranched; black, anteiso-branched. Middle panels: turquoise, fatty acids; light gray, plasmalogens; dark blue, fatty aldehydes. Right panels: yellow, long-chain >C_16_; light blue, short-chain <C_16_.

These observations indicated, not unexpectedly, that the cells restructured their outer cell envelope in response to a temperature much below T_OPT_. We next determined the fatty acid composition of the cytoplasmic membrane of *T. kivui* DSM2030 and Adpt45_67 at 66°C and 50°C (Figure 6 and Supplementary Figure S2). In the type strain, the major fatty acids with the largest fractions were i-C_15:0_, *i*-C_17:0_, and C_16:0_, with branched fatty acids representing ~93% of total fatty acids. At the first glance, the membrane fatty acid composition of Adpt45_67 appears to be extremely similar; it also contains high amounts of the same fatty acids. A closer look revealed a much higher fraction of dimethyl acetals, indicative of plasmalogens (~37% vs. 17% in the type strain at 66°C, Figure 6B), including the plasmalogens *i*-C_16:0_ P (at 66°C) and *a*-C_15:0_ P (at 50°C) that were only detected in Adpt45_67. A surprising observation was the absence of unsaturated fatty acids in the fatty acid content of both strains, except for the type strain at 60°C, where a small amount of C(18:1) w7c was detected. In general, the fatty acid composition varies between bacterial species and can even be strain-specific, with nutrient availability and growth phase also showing an influence (Suutari and Laakso, 1992; Siliakus et al., 2017). Accordingly, bacteria have a number of options to adapt their fatty acid composition and thus membrane fluidity to environmental conditions. For the genus *Bacillus* and *Clostridia*, it is known that the major fatty acids are branched chained and that the regulation of fatty acid composition is mainly driven by the type of branching and chain length (Chan et al., 1971; Oshima and Miyagawa, 1974; Sikorski et al., 2008). When comparing thermophilic with mesophilic representatives, the utilization of iso-branched FAs in favor of anteiso-branched FAs was shown to decrease with decreasing temperature (Suutari and Laakso, 1992; Sikorski et al., 2008; Siliakus et al., 2017). This change in ratio was also observed between the type strain and Adpt45_67 as well as the reduction in chain length. Growth at a suboptimal temperature of 50°C resulted in a general shortening of fatty acids in the profiles of the strains studied, as expected, with an increase in C_11_ to C_15_ fatty acids and a decrease in C_16_ and C_17_ fatty acids. Notable among these were a sharp decrease in the plasmalogen *i*-C_17:0_ P (from 11.4 to 3.5%) and a significant increase in *i*-C_15:0_ (from 42.9 to 58.8%) in the type strain. Since their overall concentration decreased at 50°C in the type strain, plasmalogens likely do not represent a short-term response to growth at suboptimal temperatures in *T. kivui*. Plasmalogens are typical components of the cell membrane of strictly anaerobic bacteria (Goldfine, 2010; Jackson et al., 2021). They are characterized by the presence of a vinyl ether linkage at the sn-1 position and an ester linkage at the sn-2 position. Plasmalogens have a lower transition temperature than their diacyl counterparts, which is why they are considered to play a role in shaping the biophysical properties of cellular membranes (Goldfine, 2010; Koivuniemi, 2017; Vítová et al., 2021). Thus, the increased use of plasmalogens could be a way for *T. kivui* to fine-tune membrane fluidity at lower temperatures. In this study, Adpt45_67 shows a significantly higher concentration of plasmalogens than the type strain. It should be noted, however, that the plasmalogens are also subject to the aforementioned reduction in chain length with decreasing temperature.

In order to elucidate the genomic changes responsible for the phenotype observed, the genome of T. *kivui* Adpt45_67 was sequenced and compared to that of the type strain (Hess et al., 2014). Altogether, 67 single-nucleotide polymorphisms were observed across the 2.397 Mbp genome (Supplementary Table S2). Thirty-three of the SNPs were also in genomes of strains adapted to other environmental stresses in our laboratory (unpublished data). Among these was a mutation (P216L; CCA → CTA) in *fabG*, a gene essential for fatty acid metabolism that encodes the 3-oxoacyl-[acyl-carrier-protein] reductase, which may have contributed to changes in fatty acid composition. Among the 34 remaining unique mutations, nine were found to be silent. Another 14 were attributed to genes with hypothetical or putative functions, leaving 11 unique, non-silent SNPs in genes or intergenic regions affecting gene expression with annotated functions (Table 1). Four of the SNPs were only found in part of the analyzed DNA, meaning that the majority of the population still contained the original base. This is likely as Adpt45_67 may be considered an adapted population, since it was not re-isolated from solid media, and therefore, the partial SNPs may be interpreted as transient toward a cold-adapted phenotype, and it will be studied whether they carry through in later generations of the ongoing ALE of *T. kivui* Adp45 at 45°C. Among those SNPs are two that affect the same transposase, TKV_c7000, indicating that this transposase may be involved in temperature response. Moreover, 5.7% of Adpt45_67 led to the mutation G28V in the alternative sigma factor H. This is of interest, since SigH has been described to be involved in several bacteria, including the Gram-positive *Corynebacterium glutamicum*, where it is involved in heat shock and oxygen stress responses (Kim et al., 2005), though the deletion of *sigH* in *Synechococcus* PCC 7002 does not affect growth at a suboptimal temperature (Inoue-Sakamoto et al., 2007). In *Staphylococcus aureus*, the gene is involved in competence for DNA uptake (Morikawa et al., 2012). Seven SNPs were found in 100% of the reads. Two of them are located in the intragenic regions and may affect the expression level of the genes encoding an ATPase and an alanine racemase, the latter of which is involved in cell wall biogenesis. Two more are located in transcriptional regulators that have relatively low expression levels at 66°C (unpublished data), but may be of importance in cold adaptation. Since *T. kivui* easily takes up and integrates environmental DNA at 66°C (Basen et al., 2018; Zeldes et al., 2023), the SNP in Cas6-1, a CRISPR RNA maturation endonuclease (Jesser et al., 2019), may modulate the ability to acquire foreign DNA as a strategy for the adaptation of the environment. Another SNP was detected in the gene encoding the potassium ATPase *kdpC* N146K (AAT → AAG). It is tempting to speculate that potassium has an influence on cold adaptation in *T. kivui* since, recently, the respective potassium uptake system has been shown to be involved in the cold adaptation of the mesophilic oligotrophic bacterium *Caulobacter crescentus* (de Araujo et al., 2021). Finally, an SNP R136T (AGG → ACG) was found in the gene of the cAMP-binding protein, Crp (TKV_c24530). Crp is a global regulator (Ma et al., 2004) that, on the one hand, may be involved in temperature response, providing Adpt45_67 a fitness advantage at lower temperatures, and that is linked to sugar uptake via PTS systems, which, on the other hand, may explain the observed weaker growth of Adpt45_67 in a defined medium.

**Table 1:** Non-silent or intergenic single-nucleotide polymorphisms (SNPs) unique to the genome of *T. kivui* Adpt45_67 (*i.e.*, not yet observed in other *T. kivui* adapted strains).

**Position (Bp)**	**SNP**	**reads with SNPs (%)**	**Amino acid change**	**Gene number (→ plus strand, ←minus strand)**	**Annotation**
268,757	C → T	100	A97V (GCG → GTG)	TKV_c02640 →	transcriptional regulator, TetR family
688,064	A → G	9.3	intergenic (+216/-54)	TKV_c07000 → / → TKV_c07010	transposase for insertion sequence element IS629/hypothetical protein
688,089	G → A	6.7	intergenic (+241/-29)	TKV_c07000 → / → TKV_c07010	transposase for insertion sequence element IS629/hypothetical protein
899,158	G → C	14.1	intergenic (-134/-42)	TKV_c09420 ← / → TKV_c09430	phosphatidylglycerophosphatase A-like protein/hypothetical protein
1,419,632	C → T	100	A116T (GCA → ACA)	TKV_c14800 ←	transcriptional regulator, TraR/DksA family
1,435,634	A → T	100	intergenic (-56/+41)	TKV_c14990 ← / ← TKV_c15000	alanine racemase domain-containing protein/membrane fusion protein
1,442,610	Δ1 bp	100	intergenic (-94/+15)	TKV_c15050 ← / ←*nadC*	ATPase associated with various cellular activities AAA_3/nicotinate-nucleotide pyrophosphorylase
1,758,229	A → C	100	N146K (AAT → AAG)	*kdpC* ←	potassium-transporting ATPase C chain
1,982,739	C → A	5.7	G28V (GGG → GTG)	*sigH* ←	RNA polymerase sigma-H factor SigH
2,348,017	G → T	100	A39E (GCG → GAG)	*cas6a* ←	CRISPR-associated endoribonuclease Cas6 1
2,372,359	C → G	100	R136T (AGG → ACG)	TKV_c24530 ←	cAMP-binding protein

In this study, only SNPs in genes with annotated function are listed (for a complete list of SNPs, see Supplementary Figure S2).

In conclusion, the genotype of *T. kivui* changed in response to prolonged incubation at 45°C in complex media at several loci that have been attributed to regulation or temperature adaptation in bacteria.

## 4. Discussion

There is an ongoing controversy about whether the first cells were (hyper)thermophilic or mesophilic. On the one hand, and from a human perspective, hot temperatures are extreme, and it seems straightforward to think that thermophiles must have evolved from mesophiles; on the other hand, ~4 billion years ago, when life arose, the Earth or at least parts of it such as the Hadean Ocean were still relatively warm (Lunine, 2006). One of the pro-arguments for a hot-start, however, is the existence of a reverse gyrase in both domains of life, Bacteria, and Archaea, which has been argued based on its phylogeny (Catchpole and Forterre, 2019), and beyond that, it might be acquired through horizontal gene transfer by either domain. It has also been suggested that the progenitor may have been a moderate thermophile that evolved at a submarine alkaline, moderately hot vent such as the Lost City hydrothermal vent field (Russell et al., 2010; Sousa et al., 2013). Moreover, the progenitor, or LUCA, the last universal ancestor of Archaea and Bacteria, was proposed to share features of recent acetogenic bacteria and methanogenic archaea (Martin and Sousa, 2016). Both acetogenesis and methanogenesis are dependent on energy conservation from the conversion of H_2_+CO_2_. Genes toward H_2_+CO_2_ utilization, the respective essential cofactor biosynthesis pathways and the terminal electron accepting pathway of acetogens and CO_2_ fixation pathway in methanogens, the Wood–Ljungdahl pathway (WLP; Ljungdahl, 1986; Wood et al., 1986), were suggested to be present in the genome of LUCA (Weiss et al., 2016). Thus, acetogens are likely ancient (Basen and Müller, 2017; Martin, 2020). It may ultimately be unresolvable whether the progenitor was a thermophile or a mesophile. Undisputable, however, thermophiles are ancient, with many archaeal and bacterial phyla at the root of phylogenetic trees (Pace, 1997; Stetter, 2006), and it is likely that at least in some cases mesophilic microorganisms evolved from thermophilic ancestors, as reported for the ancient bacterial phylum *Thermotogales* (Zhaxybayeva et al., 2009) and for mesophilic *Methanococcus* species (Lecocq et al., 2021) based on phylogenetic analyses. It still remains intriguing how this evolution happened since it required a stepwise molecular adaptation based on modifications to the genome.

While the aforementioned studies are based on comparative and phylogenetic analyses of genomes, the ALE of a thermophile toward a lower T_OPT_ has never been tested experimentally, to the best of our knowledge. This triggered us to grow and transfer the acetogen *Thermoanaerobacter kivui* at 45°C, a temperature 5–10°C above its T_MIN_ and 21°C below its T_OPT_. The choice fell on an acetogen since acetogenesis—as thermophily—is an ancient trait (Basen and Müller, 2017), as described above. After ~180 generations/67 transfers at 45°C, the *T. kivui* population (strain Adpt45_67) was surprisingly not better adapted to 45°C, but to temperatures 5–10 K below the T_OPT_ (55–60°C). This is somewhat in line with an observation and hypothesis published over 30 years ago by Jürgen Wiegel. He reported on a temperature plateau below the T_OPT_ of certain anaerobic thermophiles, including *Thermoanaerobacter* species, allowing them to relatively quickly adapt to slightly lower temperatures. Moreover, their growth temperature (tolerance) range is large (35–78°C) compared to that of many mesophiles (Onyenwoke and Wiegel, 2015). Considering a scenario in which single *Thermoanaerobacter* sp. cells were suddenly exposed to a suboptimal temperature—in the ancient world or recently—their capability to cope at much lower temperatures may have prevented their rapid extinction in the environment, and that may equally hold true for other thermophiles with a broad temperature span (Wiegel, 1990). In that regard, it is worth noticing that there is increasing evidence for thermophiles in cold habitats far below their T_MIN_, such as Arctic sediments, soils, seawater, and even ice (Milojevic et al., 2022). The perseverance of thermophiles in arctic habitats correlates well with their broad temperature range. These populations are likely transported from populations in warmer habitats such as deep sediments, subsurface petroleum reservoirs, or ocean ridges (Hubert et al., 2009), and they may resemble ancestral thermophiles in their development toward mesophile.

Considering the evolution of a mesophile to a thermophile, mesophiles possess a narrower temperature range, ~30–35°C (Wiegel, 1990). Particularly, they face the challenge that temperatures slightly exceeding the upper-temperature limit (T_MAX_) may quickly lead to protein denaturation. Nonetheless, ALE experiments in mesophiles toward higher T_OPT_ have been carried out, with mixed results. A successful evolution of *E. coli* in a continuous system with gradually increasing temperatures (from 44°C to 49.7°C) was reported (Blaby et al., 2012). The resulting strain had a significantly increased T_OPT_ of 46°C, demonstrating that (moderate) thermophiles may have evolved from mesophilic ancestors. The adapted strain carried 31 single-nucleotide substitutions, among these putative critical mutations in the glycerol facilitator gene *glpF* and in the fatty acid desaturase/isomerase *fabA*. Interestingly, a mutation in the *kdpD* gene was recognized, a sensor kinase associated with the potassium ATPase gene *kdpC*, which we observed to carry a mutation. In another experiment, sequential transfer of a mutator strain *Zymomonas mobilis* and of *E. coli* with stepwise temperature increase for ~400 and 550 generations, respectively, resulted in an increased T_MAX_ of 2–3 K (to 41°C and to 47°C), with cells dying if the temperature was further increased (Kosaka et al., 2019). The number of mutations observed in the thermally adapted strains was surprisingly small (24 and 9, respectively), in the same range as observed in this study. While most studies of thermal adaptations aimed to increase T_OPT_ or T_MAX_, often in the context of adaptation to climate change or toward a biotechnological application, recently, a community approach was carried out to evolve *Escherichia coli* (T_OPT_ 37°C) and *Saccharomyces cerevisiae* (T_OPT_ 30°C) to 20°C and 15°C, respectively, ~15 K below their T_OPT_ (Strauss et al., 2019). More than 20 groups from all over the world tried different approaches for a thermal adaptation of the two organisms to a suboptimal temperature. Most ALE approaches were successful; however, the two organisms behaved differently. In *E. coli*, the most common phenotype was a decrease in the lag phase, while in *S. cerevisiae*, the yield at the lower temperature increased. Improvement of growth rate was not observed or only to a limited extent, similar to what we observed with *T. kivui* Adpt45_67; however, we did not observe a lag phase in *T. kivui* of >1 h when transferred to 45°C. Unfortunately, the authors do not report on whether T_OPT_ or T_MIN_ were affected.

Microorganisms persevering in an unfavorable environment, even if they grow at low rates, allow time for a population to “gather” mutations, some of which are selected for growth and adaptation to the unfavorable environment. Adpt45_67 obviously is not yet adapted for better growth at 45°C, but sustains at 39°C, and, compared to the wild type, showed a T_OPT_ shifted to 60°C. It is not clear yet, whether this adaptation halts on the plateau of 60–55°C (Wiegel, 1990), or whether more generations at 45°C will further shift the T_OPT_ and finally increase the growth rate at 45°C. In that case, the observed genotype here may be interpreted as transient. Only a few unique and non-silent mutations were identified (Table 1), with some of them potentially related to temperature adaptation or gene regulation. Targeted point mutations may identify which of these SNPs are essential to the adapted phenotype. Continuation of the ALE may identify further mutations and allow insights into their order of occurrence. We conclude that *Thermoanaerobacter* and many thermophiles are well prepared to survive or even thrive at ambient temperatures (Wiegel, 1990), which may have facilitated not only a slight transition of the T_OPT_ but a complete thermophile-to-mesophile transition over large evolutionary time scales.

The evolution of thermal adaptation has been linked to a generally different amino acid composition. From a pan-genome analysis, it is evident that the evolution of mesophilic *Methanococcus* sp. from thermophilic ancestors was directly linked to the replacement of lysine and an increase in the percentage of threonine, glutamine, serine, aspartate, and asparagine (Lecocq et al., 2021). The authors conclude that amino acid replacement via single mutations rather than horizontal gene transfer (HGT) events caused the adaptation to the lower temperature. This reflects, however, large evolutionary time scales, and cannot explain adaptations to slightly lower temperatures in a shorter time period as observed here. On a shorter time scale, it may be conceivable that thermophiles profit from HGT events in adaptation to lower temperatures. *T. kivui*, the organism used in this study, has recently been shown to efficiently take up and integrate foreign DNA from a laboratory environment into its genome, which allowed the strain to adapt to a certain medium type (Zeldes et al., 2023).

Further attempts to adapt thermophiles to lower temperatures in the laboratory are warranted to elucidate whether the observed shift toward a lower T_OPT_ can be reproduced or increased beyond the temperature plateau and whether growth also improves at the lower temperature end. A difficulty that we encountered was certainly the low growth rates at much lower temperatures, which decrease the number of generations (and thus the number of possible mutations) in ALE experiments. Nonetheless, experimental approaches toward a cold adaptation of thermophiles will be essential to ultimately prove considerations from genome comparisons. It will be of interest to see whether the ALE of thermophiles toward a lower T_OPT_ will lead to an increase in the GC content (Hu et al., 2022) or a change in the amino acid pattern (Sauer and Wang, 2019; Lecocq et al., 2021) in the long term. In the short term, these experiments may resolve the order of single evolutive events of particular importance, such as single gene (e.g., transcriptional regulators) inactivation by point mutations or HGT events (in case DNA of mesophiles is supplied), and enable the study of the respective phenotypic effects in single organisms, such as in the acetogen *T. kivui*.

## Data availability statement

The datasets presented in this study can be found in online repositories. The names of the repository/repositories and accession number(s) can be found below: https://www.ncbi.nlm.nih.gov/sra/, SRR25301688.

## Author contributions

ML: Data curation, Formal analysis, Investigation, Methodology, Validation, Visualization, Writing—original draft. CP: Validation, Writing—original draft, Data curation, Formal analysis, Investigation, Methodology, Visualization. BZ: Investigation, Validation, Data curation, Formal analysis, Methodology, Visualization, Writing—review and editing. AP: Resources, Software, Writing—review and editing, Data curation, Formal analysis, Investigation. RD: Writing—review and editing, Resources, Software. MB: Writing—original draft, Validation, Conceptualization, Funding acquisition, Project administration, Resources, Supervision.
