# Supplementary material for: Adaptive laboratory evolution of a thermophile toward a reduced growth temperature optimum
Source: Front Microbiol. 2023 Oct 12;14:1265216. doi: 10.3389/fmicb.2023.1265216 (PMC10601643; doi:10.3389/fmicb.2023.1265216)
Supplement: Supplementary file 1 [file Data_Sheet_1.docx]

Supplementary Material

for the manuscript

**Adaptive laboratory evolution of a thermophile towards a reduced growth temperature optimum**

**by**

**Maria Lehmann^1#^, Christoph Prohaska^1#^, Benjamin Zeldes^1^, Anja Poehlein^2^, Rolf Daniel^2^ and Mirko Basen^1*^**

^1^Microbiology, University of Rostock, Institute of Biological Sciences, Albert-Einstein Str. 3, 18059 Rostock, Germany

^2^Genomic and Applied Microbiology & Göttingen Genomics Laboratory, Georg-August University, Göttingen, Germany

# These authors contributed equally to this work

*** Correspondence:**

Mirko Basen

[mirko.basen@uni-rostock.de](mailto:mirko.basen@uni-rostock.de)

Keywords: adaptive laboratory evolution, origin of Life, cold adaptation, acetogens, thermophiles, *Thermoanaerobacter kivui*,

# Supplementary Figures and Tables

## Supplementary Figures


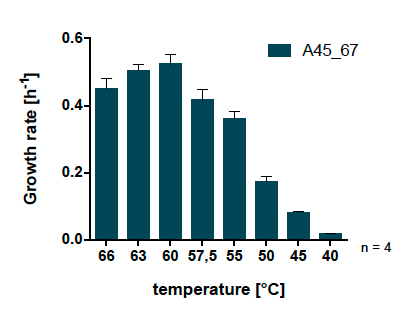


**Supplementary Figure 1.** Growth rates of *T. kivui* Adpt56_67 at different temperatures (2.5 K resolution). Cells were grown in 50 ml complex medium in 100 ml serum bottles with 25 mM glucose as carbon source. The error indicated shows the standard deviation from four biological replicates.


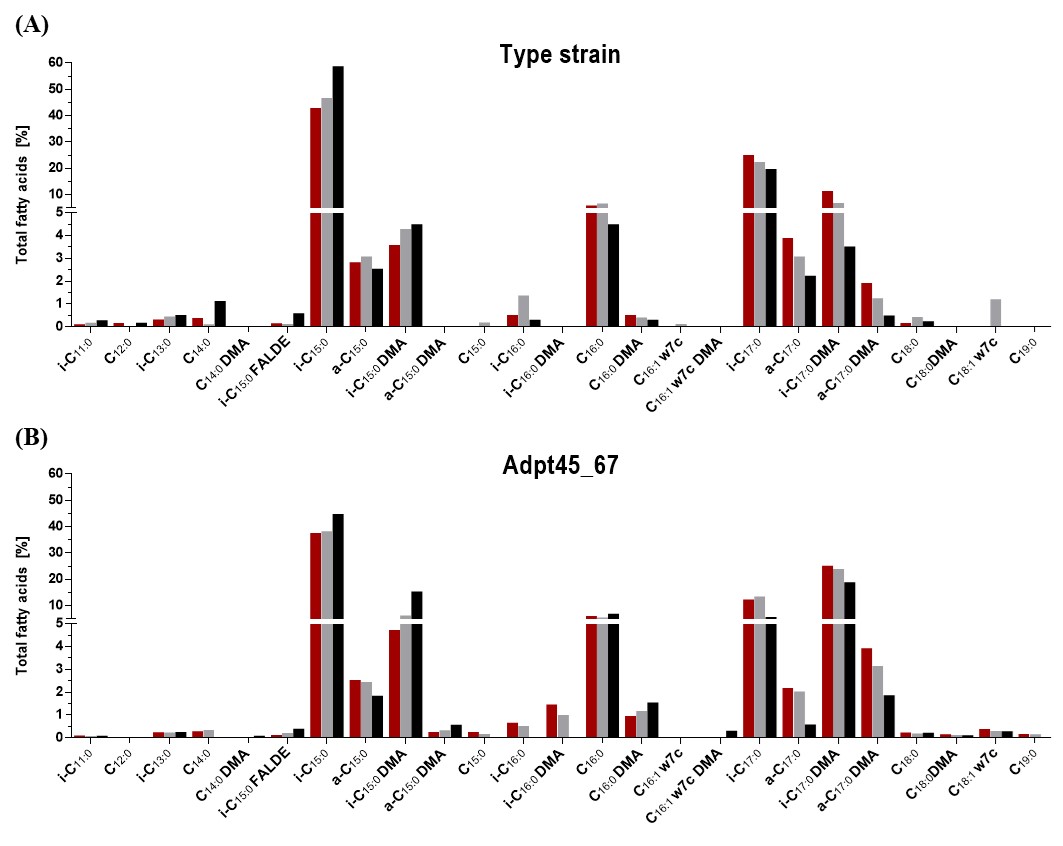


**Supplementary Figure 2**. Detailed total fatty acid composition of **(A)** *T. kivui* DSM2030 (type strain) and **(B)** Adpt45_67, grown in complex medium with 25 mM glucose at optimal (66°C, 60°C) and suboptimal (50°C) temperature to an OD_600_ of approx. 1. Red, 66°C; grey, 60°C; black, 50°C. i, iso-branched; a, anteiso-branched; DMA, plasmalogen; FALDE, fatty aldehyde.

**

**

**Supplementary Figure 3.** Full trajectory of *T. kivui* populations serially passaged (after Strauss *et al.,* 2019) at 45 °C. Five subpopulations of *T. kivui* were incubated for 24 hours in complex medium containing 25 mM glucose as a carbon source. The subpopulation with the highest increase in OD_600_ was used to generate five new subpopulations in fresh medium. The transfers were performed in the exponential growth phase (see Fig. 3).

**Supplementary Table 1.** Specific growth rates of *T. kivui* DSM2030 (type strain), Adpt_45_67 at different temperatures. Cells were grown in 50 ml complex medium in 100 ml serum bottles with 25 mM glucose as carbon source (n=4 ± standard deviation, SD) (corresponding to graphs presented in Fig. 3).

| Temperature | WT | | Adpt_45_67 | |
| --- | --- | --- | --- | --- |
| [°C] | µ [h^-1^] | SD | µ [h^-1^] | SD |
| 66 | 0.449 | 0.008 | 0.449 | 0.031 |
| 60 | 0.391 | 0.009 | 0.523 | 0.029 |
| 55 | 0.297 | 0.006 | 0.360 | 0.022 |
| 50 | 0.183 | 0.005 | 0.173 | 0.016 |
| 45 | 0.104 | 0.003 | 0.080 | 0.004 |
| 40 | 0.033 | 0.001 | 0.017 | 0.003 |

**Supplementary Table 2** Complete list of single nucleotide polymorphisms in the genome of *T. kivui* Adpt45_67 vs. *T. kivui* DSM 2030.

| **Position (Bp)** | **SNP** | **reads with SNPs (%)** | **Amino acid change** | **Gene number (→ plus strand, ← minus strand)** | **Annotation** |
| --- | --- | --- | --- | --- | --- |
| 255,372 | Δ1 bp | 100 % | coding (102/264 nt) | TKV_c02510 ← | hypothetical protein |
| 260,05 | A→T | 100 % | L3F (TTA→TTT) | TKV_c02590 → | hypothetical protein |
| 268,757 | C→T | 100 % | A97V (GCG→GTG) | TKV_c02640 → | transcriptional regulator, TetR family |
| 285,041 | G→C | 100 % | W100S (TGG→TCG) | pit → | low‑affinity inorganic phosphate transporter Pit |
| 341,995 | T→C | 100 % | *207Q (TAG→CAG) | cbiQ → | cobalt ABC transporter, inner membrane subunit CbiQ |
| 390,527 | T→C | 6.4% | D18D (GAT→GAC) | TKV_c03940 → | radical SAM domain‑containing protein |
| 542,8 | (22-bp)2→1 | 100 % | intergenic (+843/‑856) | TKV_c05490 → / → TKV_c05510 | 2‑polyprenylphenol hydroxylase‑like oxidoreductase/ferredoxin‑dependent glutamate synthase |
| 575,94 | G→A | 100 % | E245K (GAA→AAA) | mtlR2 → | transcriptional regulator MtlR |
| 577,692 | G→C | 100 % | G41A (GGA→GCA) | TKV_c05840 → | phosphotransferase system cellobiose‑specific component IIB |
| 676,845 | C→T | 100 % | N44N (AAC→AAT) | TKV_c06880 → | hypothetical protein |
| 687,398 | A→G | 12.1% | K150K (AAA→AAG) | TKV_c07000 → | transposase for insertion sequence element IS629 |
| 687,404 | T→C | 11.9% | G152G (GGT→GGC) | TKV_c07000 → | transposase for insertion sequence element IS629 |
| 687,413 | C→T | 15.1% | Y155Y (TAC→TAT) | TKV_c07000 → | transposase for insertion sequence element IS629 |
| 687,422 | C→T | 17.4% | A158A (GCC→GCT) | TKV_c07000 → | transposase for insertion sequence element IS629 |
| 688,064 | A→G | 9.3% | intergenic (+216/‑54) | TKV_c07000 → / → TKV_c07010 | transposase for insertion sequence element IS629/hypothetical protein |
| 688,089 | G→A | 6.7% | intergenic (+241/‑29) | TKV_c07000 → / → TKV_c07010 | transposase for insertion sequence element IS629/hypothetical protein |
| 728,674 | G→T | 100 % | E3* (GAA→TAA) | TKV_c07390 → | hypothetical protein |
| 759,158 | Δ1 bp | 100 % | intergenic (+251/‑273) | TKV_c07880 → / → TKV_c07890 | hypothetical protein/hypothetical protein |
| 761,442 | Δ1 bp | 100 % | intergenic (+572/‑276) | TKV_c07910 → / → TKV_c07920 | hypothetical protein/bacteriocin class II, amylovorin‑like protein |
| 831,064 | G→A | 100 % | L153F (CTC→TTC) | TKV_c08720 ← | hypothetical protein |
| 831,5 | Δ1 bp | 100 % | coding (21/1152 nt) | TKV_c08720 ← | hypothetical protein |
| 881,043 | Δ1 bp | 100 % | coding (1719/1854 nt) | TKV_c09200 → | hypothetical protein |
| 899,158 | G→C | 14.1% | intergenic (‑134/‑42) | TKV_c09420 ← / → TKV_c09430 | phosphatidylglycerophosphatase A‑like protein/hypothetical protein |
| 899,258 | C→T | 30.4% | T20M (ACG→ATG) | TKV_c09430 → | hypothetical protein |
| 899,261 | G→A | 30.9% | R21K (AGA→AAA) | TKV_c09430 → | hypothetical protein |
| 899,271 | A→G | 35.4% | R24R (AGA→AGG) | TKV_c09430 → | hypothetical protein |
| 899,587 | Δ1 bp | 27.8% | coding (227/249 nt) | TKV_c09440 → | hypothetical protein |
| 899,602 | T→C | 23.6% | L81S (TTA→TCA) | TKV_c09440 → | hypothetical protein |
| 913,68 | A→G | 100 % | Q44R (CAA→CGA) | TKV_c09570 → | hypothetical protein |
| 913,682 | G→A | 100 % | D45N (GAC→AAC) | TKV_c09570 → | hypothetical protein |
| 913,696 | T→A | 100 % | S49R (AGT→AGA) | TKV_c09570 → | hypothetical protein |
| 913,718 | A→G | 100 % | K57E (AAA→GAA) | TKV_c09570 → | hypothetical protein |
| 913,725 | G→A | 100 % | *59* (TGA→TAA) | TKV_c09570 → | hypothetical protein |
| 913,74 | C→G | 100 % | intergenic (+14/‑53) | TKV_c09570 → / → TKV_c09580 | hypothetical protein/hypothetical protein |
| 913,759 | G→A | 100 % | intergenic (+33/‑34) | TKV_c09570 → / → TKV_c09580 | hypothetical protein/hypothetical protein |
| 913,761 | G→A | 100 % | intergenic (+35/‑32) | TKV_c09570 → / → TKV_c09580 | hypothetical protein/hypothetical protein |
| 913,791 | C→T | 100 % | intergenic (+65/‑2) | TKV_c09570 → / → TKV_c09580 | hypothetical protein/hypothetical protein |
| 913,798 | A→G | 100 % | G2G (GGA→GGG) | TKV_c09580 → | hypothetical protein |
| 913,803 | C→T | 100 % | S4L (TCA→TTA) | TKV_c09580 → | hypothetical protein |
| 1,062,261 | A→G | 100 % | V557V (GTA→GTG) | TKV_c10980 → | vitamin B12 dependent methionine synthase |
| 1,140,176 | G→A | 100 % | S71N (AGC→AAC) | TKV_c11810 → | hypothetical protein |
| 1,320,589 | G→A | 100 % | P216L (CCA→CTA) | fabG2 ← | 3‑oxoacyl‑[acyl‑carrier‑protein] reductase FabG |
| 1,419,632 | C→T | 100 % | A116T (GCA→ACA) | TKV_c14800 ← | transcriptional regulator, TraR/DksA family |
| 1,435,634 | A→T | 100 % | intergenic (‑56/+41) | TKV_c14990 ← / ← TKV_c15000 | alanine racemase domain‑containing protein/membrane fusion protein |
| 1,442,610 | Δ1 bp | 100 % | intergenic (‑94/+15) | TKV_c15050 ← / ← nadC | ATPase associated with various cellular activities AAA_3/nicotinate‑nucleotide pyrophosphorylase |
| 1,559,965 | T→C | 100 % | N35D (AAT→GAT) | gap ← | glyceraldehyde‑3‑phosphate dehydrogenase Gap |
| 1,587,697 | Δ1 bp | 100 % | coding (1476/1644 nt) | TKV_c16590 ← | AAA ATPase |
| 1,620,604 | G→T | 100 % | A53E (GCG→GAG) | TKV_c16910 ← | hypothetical protein |
| 1,758,229 | A→C | 100 % | N146K (AAT→AAG) | kdpC ← | potassium‑transporting ATPase C chain |
| 1,887,564 | C→G | 100 % | intergenic (‑11/+416) | hydC ← / ← TKV_c19610 | electron bifurcating hydrogenase subunit HydC/putative nickel‑responsive regulator |
| 1,982,739 | C→A | 5.7% | G28V (GGG→GTG) | sigH ← | RNA polymerase sigma‑H factor SigH |
| 1,982,829 | G→A | 100 % | intergenic (‑8/+73) | sigH ← / ← TKV_c20750 | RNA polymerase sigma‑H factor SigH/hypothetical protein |
| 2,117,454:1 | +T | 100 % | intergenic (‑159/+749) | TKV_c22160 ← / ← argH | UBA/THIF‑type NAD/FAD binding protein/argininosuccinate lyase ArgH |
| 2,131,088 | T→G | 100 % | intergenic (+358/‑12) | TKV_c22250 → / → adh | transposase IS1161/NADP‑dependent isopropanol dehydrogenase Adh |
| 2,134,425:1 | +G | 100 % | coding (1208/1224 nt) | nfnB → | transhydrogenase subunit B |
| 2,149,190 | G→A | 100 % | S174N (AGT→AAT) | insK → | putative transposase InsK for insertion sequence element IS150 |
| 2,149,272 | C→T | 100 % | D201D (GAC→GAT) | insK → | putative transposase InsK for insertion sequence element IS150 |
| 2,149,284 | G→A | 100 % | Q205Q (CAG→CAA) | insK → | putative transposase InsK for insertion sequence element IS150 |
| 2,149,290 | T→A | 100 % | T207T (ACT→ACA) | insK → | putative transposase InsK for insertion sequence element IS150 |
| 2,149,690 | T→C | 100 % | intergenic (+121/+80) | insK → / ← TKV_c22490 | putative transposase InsK for insertion sequence element IS150/hypothetical protein |
| 2,149,742 | T→C | 100 % | intergenic (+173/+28) | insK → / ← TKV_c22490 | putative transposase InsK for insertion sequence element IS150/hypothetical protein |
| 2,252,722 | G→C | 100 % | *436S (TGA→TCA) | TKV_c23430 → | amino acid permease |
| 2,270,989 | Δ271 bp | 100 % | intergenic (‑2880/+3043) | TKV_c23580 ← / ← cas2 | hypothetical protein/CRISPR‑associated endoribonuclease Cas2 |
| 2,346,306 | Δ67 bp | 100 % | intergenic (‑1685/+1005) | TKV_c24260 ← / ← cas6a | bacterial capsule synthesis protein/CRISPR‑associated endoribonuclease Cas6 1 |
| 2,347,980 | T→A | 100 % | K51N (AAA→AAT) | cas6a ← | CRISPR‑associated endoribonuclease Cas6 1 |
| 2,348,017 | G→T | 100 % | A39E (GCG→GAG) | cas6a ← | CRISPR‑associated endoribonuclease Cas6 1 |
| 2,372,359 | C→G | 100 % | R136T (AGG→ACG) | TKV_c24530 ← | cAMP‑binding protein |
